# Supplementary material for: An Experimental Investigation of the Mechanical Performance of EPS Foam Core Sandwich Composites Used in Surfboard Design
Source: Polymers (Basel). 2023 Jun 16;15(12):2703. doi: 10.3390/polym15122703 (PMC10304318; doi:10.3390/polym15122703)
Supplement: Supplementary file 1 [file polymers-15-02703-s001.zip › polymers-2413300-supplementary.pdf]

supplementary

An Experimental Investigation of the Mechanical Performance of EPS Foam Core Sandwich Composites Used in Surfboard Design

Sam Crameri [1,*], Filip Stojcevski [1,2] and Clara Usma-Mansfield [1]

[1] School of Engineering, Deakin University, Waurn Ponds, *VIC* 3216, Australia
[2] Institute for Frontier Materials, Deakin University, Waurn Ponds, *VIC* 3216, Australia
* Correspondence: slcramer@deakin.edu.au; Tel.: +61433755945

Table S1. Medium density foam results.

Code			M-1EG		M-2EG		M-90CF		M-45CF		M-PET	
			Mean	± SD	Mean	± SD	Mean	± SD	Mean	± SD	Mean	± SD
Fibre Weight Fraction	(W_f)		0.47		0.54		0.50		0.49		0.26	
Max Flexural Strength	(σ_{max})	MPa	1.96	0.57	4.07	0.27	4.92	0.60	3.32	0.64	3.57	0.42
Flexural Modulus	(E_f)	GPa	0.84	0.06	1.44	0.07	2.28	0.12	1.16	0.05	0.53	0.03
Flexural Yield Strength	(σ_y)	MPa	1.75	0.54	4.05	0.28	4.91	0.60	3.30	0.65	3.54	0.41
Flexural Yield Strain	(ε_y)	mm/mm	0.00407	0.00057	0.00483	0.00031	0.00416	0.00032	0.00486	0.00059	0.00871	0.00102
Material Toughness	(UT)	J/m³	0.0043	0.001	0.0137	0.002	0.0147	0.004	0.0109	0.003	0.0153	0.002
Shear Modulus	(G_S)	GPa	0.108	0.026	0.303	0.032	0.358	0.073	0.550	0.160	0.166	0.009
Shear Yield Strength	(τ_y)	MPa	1.36	0.19	3.11	0.13	2.87	0.06	3.99	0.47	2.07	0.07
Shear Yield Strain	(γ_Y)	mm/mm	0.0174	0.0026	0.0178	0.0008	0.0177	0.0016	0.0148	0.0017	0.0204	0.0012
Shear Toughness	(ST)	J/m³	0.0412	0.0088	0.0459	0.0083	0.0923	0.0044	0.0789	0.0076	0.0661	0.0012
Fracture Toughness	(K_{IC})	J/m³	0.0116	0.0015	0.0326	0.0060	0.0366	0.0069	0.0512	0.0079	0.0246	0.0115
Tensile Strength	(F_{tu})	MPa	7.755	0.986	11.391	0.831	31.922	3.230	4.541	0.298	4.383	0.495
Tensile Modulus	(E_T)	GPa	0.287	0.035	0.586	0.024	1.147	0.118	0.287	0.025	0.163	0.016

Table S1: High density foam results

Code			H-1EG		H-2EG		H-90CF		H-45CF		H-PET	
			**Mean**	**± SD**	**Mean**	**± SD**	**Mean**	**± SD**	**Mean**	**± SD**	**Mean**	**± SD**
**Fibre Weight Fraction**	**(W_f)**		0.46		0.51		0.52		0.50		0.27	
**Max Flexural Strength**	**(σ_{max})**	**MPa**	1.56	0.12	2.56	0.34	2.62	0.72	2.29	0.31	1.96	0.32
**Flexural Modulus**	**(E_f)**	**GPa**	0.74	0.03	0.99	0.10	1.26	0.08	0.90	0.04	0.45	0.03
**Flexural Yield Strength**	**(σ_y)**	**MPa**	1.53	0.12	2.47	0.33	2.46	0.65	2.21	0.32	1.93	0.32
**Flexural Yield Strain**	**(ε_y)**	**mm/mm**	0.00407	0.00016	0.00448	0.00014	0.00392	0.00022	0.00447	0.00039	0.00639	0.00077
**Material Toughness**	**(UT)**	**J/m^3**	0.0045	0.001	0.0118	0.003	0.0114	0.002	0.0089	0.001	0.0077	0.002
**Shear Modulus**	**(G_S)**	**GPa**	0.121	0.019	0.335	0.040	0.273	0.046	0.623	0.097	0.110	0.014
**Shear Yield Strength**	**(τ_y)**	**MPa**	1.32	0.08	2.91	0.15	2.04	0.35	2.84	0.38	1.60	0.05
**Shear Yield Strain**	**(γ_y)**	**mm/mm**	0.0139	0.0013	0.0171	0.0009	0.0145	0.0039	0.0101	0.0027	0.0189	0.0006
**Shear Toughness**	**(ST)**	**J/m^3**	0.0322	0.0039	0.0746	0.0102	0.0748	0.0049	0.0631	0.0074	0.0515	0.0009
**Fracture Toughness**	**(K_{IC})**	**J/m^3**	0.0049	0.0008	0.0232	0.0018	0.0197	0.0029	0.0297	0.0061	0.0186	0.0037
**Tensile Strength**	**(F_{tu})**	**MPa**	6.017	0.660	6.017	0.661	35.584	1.811	3.366	0.463	4.012	0.467
**Tensile Modulus**	**(E_T)**	**GPa**	0.271	0.009	0.755	0.013	1.208	0.081	0.340	0.040	0.125	0.007
